# Onset and remission of common mental disorders among adults living in temporary housing for three years after the triple disaster in Northeast Japan: comparisons with the general population

**DOI:** 10.1186/s12889-020-09378-x

**Published:** 2020-08-20

**Authors:** Norito Kawakami, Maiko Fukasawa, Kiyomi Sakata, Ruriko Suzuki, Hiroaki Tomita, Harumi Nemoto, Seiji Yasumura, Hirooki Yabe, Naoko Horikoshi, Maki Umeda, Yuriko Suzuki, Haruki Shimoda, Hisateru Tachimori, Tadashi Takeshima, Evelyn J. Bromet

**Affiliations:** 1 Department of Mental Health, Graduate School of Medicine, The University of Tokyo, 7-3-1 Hongo Bunkyo-ku, Tokyo, 113-0033 Japan; 2 Department of Hygiene and Preventive Medicine, Iwate Medical University School of Medicine, 1-1-1 Idaidori, Yahaba-cho, Shiwa-gun, Iwate, 028-3694 Japan; 3 Department of Disaster Psychiatry, International Research Institute of Disaster Science (IRIDeS), Tohoku University, 2-1 Seiryo-machi, Aoba-ku, Sendai, Miyagi 980-8573 Japan; 4 Fukushima Medical University School of Medicine, 1 Hikariga-oka, Fukushima, Fukushima, 960-1295 Japan; 5 Research Institute of Nursing Care for People and Community, University of Hyogo, 13-71 Kitaoji, Akashi, Hyogo 673-8588 Japan; 6 National Institute of Mental Health, National Center of Neurology and Psychiatry, 4-1-1 Ogawa-Higashi, Kodaira, Tokyo, 187-8553 Japan; 7 Department of Clinical Epidemiology, Translational Medical Center, National Center of Neurology and Psychiatry, 4-1-1 Ogawa-Higashi, Kodaira, Tokyo, 187-8553 Japan; 8 Kawasaki City Center for Mental Health and Welfare, 1 Miyamoto-cho, Kawasaki-ku, Kawasaki, Kanagawa 210-8577 Japan; 9 Department of Psychiatry, Stony Brook University, 100 Nicolls Road, Stony Brook, NY 11794 USA

**Keywords:** Natural disasters, Fukushima nuclear accident, Refugees, Incidence, Help-seeking behavior

## Abstract

**Background:**

People living in temporary housing for long periods after a disaster are at risk of poor mental health. This study investigated the post-disaster incidence and remission of common mental disorders among adults living in temporary housing for the 3 years following the 2011 Great East Japan Earthquake.

**Methods:**

Three years after the disaster, face-to-face interviews were conducted with 1089 adult residents living in temporary housing in the disaster area, i.e., the shelter group, and a random sample of 852 community residents from non-disaster areas of East Japan. The World Health Organization Composite International Diagnostic Interview was used to diagnose DSM-IV mood, anxiety, and alcohol use disorders. Information on demographic variables and disaster experiences was also collected.

**Results:**

Response rates were 49 and 46% for the shelter group and the community residents, respectively. The incidence of mood/anxiety disorder in the shelter group was elevated only in the first year post-disaster compared to that of the general population. The rate of remission for mood and anxiety disorders was significantly lower in the shelter group than in the community residents. The proportion seeking medical treatment was higher in the shelter group.

**Conclusions:**

The onset of common mental disorders increased in the first year, but then levelled off in the following years among residents in temporary housing after the disaster. Remission from incident post-disaster mental disorders was slower in the shelter group than in the general population. Post-disaster mental health service could consider the greater incidence in the first year and prolonged remission of mental disorders among survivors with a long-term stay in temporary housing after a disaster.

## Background

Natural disasters, such as earthquakes and tsunamis, have a considerable impact on the onset and recurrence of mental disorders [1–5]. Previous studies reported a high prevalence of depressive disorders, anxiety disorder [6], and post-traumatic stress disorder (PTSD) [4]. These prevalence rates were 3–8 times greater compared to non-affected or less-affected populations [4, 7–11]. On the other hand, prevalence of alcohol use disorders usually did not increase after natural disasters [6, 12–14]. Some studies reported that post-disaster prevalence of PTSD sharply declined over time [11, 15], while others found the prevalence to remain high years later [16]. Much less attention has been paid to incidence rates of mood, anxiety, and substance use disorders among those with no prior history of psychopathology. In addition, far fewer studies have examined rates of remission, especially from incident post-disaster mental disorders. We are aware of no study that compared change in mental health status during the post-disaster period between disaster survivors and a community population, or factors associated with remission from post-disaster mental disorders. A key predictor of post-disaster mental health is relocation [17–21]. An analysis of data from the WHO World Mental Health Surveys reported that respondents displaced from their homes had a 6.2 times higher prevalence of common mental disorders [22]. However, most studies investigated only the short-term (e.g., within 1 year) impact of relocation.

The Great East Japan Earthquake of March 11, 2011 was a triple disaster involving an earthquake, tsunami, and meltdowns at the Fukushima Dai’ichi nuclear plant, that occurred in coastal areas of three prefectures of the Tohoku region (Iwate, Miyagi, and Fukushima) of Japan. During the disaster and its aftermath, the prolonged stay of survivors in temporary housing became a huge social and public health concern. About 220,000 residents were evacuated from their homes. By 2014, a total of 96,519 still lived in temporary housing. Relocation was consistently reported as an important factor for poor mental health among survivors of the earthquake [23]. In addition, poorer mental health was found among survivors who stayed for several years in prefabricated temporary housing [24, 25]. A recent study found that depressive symptoms among persons who were moved into prefabricated housing increased two-fold compared to residents remaining in their own homes 2.5 years after the disaster [26]. Psychological distress was particularly higher among survivors who lived in temporary housing for more than 4 years [27]. Our previous study observed an increase in suicide ideation in the first year, but not in the second or third year among survivors living in temporary housing [28]. However, it is still unclear if poorer mental health among survivors who lived longer in temporary housing is attributable to an initial increase of common mental disorders attributable to the disaster, a gradual increase in the incidence in succeeding years due to hardship in staying in temporary housing, or delayed remission from post-disaster common mental disorders. The role of medical treatment for mental disorders after the disaster in explaining poor mental health status of the survivors in temporary housing is also not known, although rates were low after previous natural disasters [29, 30].

The objectives of the present study were three-fold. First, we investigated the incidence of post-disaster DSM-IV mental disorders among relocated survivors residing in temporary housing (i.e, the shelter group) for 3 years in disaster areas of the Great East Japan Earthquake, comparing with community residents in East Japan. Second, we investigated the remission and use of medical services for of new-onset post-disaster mental disorders in the shelter group comparing with the community residents. Third, we also conducted regression analyses of the incidence and remission of post-disaster mental disorders on disaster experiences and demographic variables. This study extends our earlier research on suicidal ideation [28] by further focusing of onset and remission of common mental and alcohol use disorders.

## Methods

### Participants

#### Shelter group

Surveys were conducted with residents living in temporary housing shelters in four municipalities in three coastal areas of the Tohoku region of Japan that were severely damaged by the March 2011 earthquake, tsunami, and nuclear power plant meltdowns. Shelters were selected based on a communication with municipalities in each area. Two shelters were selected in one city of Iwate prefecture (pre-disaster population ~ 23,000) which lost 7.8% of its population in the tsunami (1601 died and 207 were lost); interviews were conducted in June–August 2014. In Miyagi prefecture, all six temporary housing residences of one town were selected. The town had a population of 20,419 in 2010; 79 died and 2 were lost due to the tsunami (0.4% in all). The survey was also conducted from June to August 2014. In Fukushima prefecture, two municipalities in the coastal area were selected. One had a population of about 71,000, with 1105 causalities (1.6%) due to the tsunami; three temporary housing residences were selected as study sites. The other had a population of about 21,000, with 541 causalities (2.6%); all residents were evacuated because of the Fukushima Dai’ichi Nuclear Plant accident. Two temporary residences were selected as study sites. The Fukushima surveys were conducted from October 2013 to February 2014. All residents aged 20 or over living in each temporary housing were approached first by a flier, followed by a visit of survey staff. We limited our sample to adults aged 20 or over, because the instrument used in the study was designed to apply to adults. The Committees of Ethics in Research of Human Subjects also allowed us to contact to obtain direct informed consent only for adults aged 20 or over.

#### General population

A total of 37 area units were randomly selected from East Japan (excluding the Kanto area), and 1850 residents aged 20–74 years (50 residents per area unit) were randomly selected based on the population registry. These areas were at least 20 km away from the disaster area and not directly damaged by the earthquake or tsunami, or forced to evacuate because of the meltdowns. These residents were contacted first by a mailed invitation latter, followed by a visit of survey staff. The survey was conducted between August and October, 2014 as part of the World Mental Health Japan Second Survey (WMHJ2) [31].

### Data collection and ethical consideration

Participants were the subjects of face-to-face, computer-assisted interviews. The instrument used in this study was the Japanese translation of the World Health Organization Composite International Diagnostic Interview (CIDI), version 3.0 [32, 33] developed for trained lay interviewers. The principal investigator (NK) obtained the permission to use the CIDI 3.0 in the surveys from the WHO World Mental Health Survey Executive Committee (see the Supplementary file). Participation was voluntary, and participants were assured of anonymity and confidentiality. Written consent was obtained from each respondent. The authors assert that all procedures contributing to this work comply with the ethical standards of the relevant national and institutional committees on human experimentation and with the Helsinki Declaration of 1975, as revised in 2008. The Committees of Ethics in Research of Human Subjects of the Graduate School of Medicine of The University of Tokyo approved the study protocol and informed consent procedure (No. 10131-(7)).

### Assessment of mental disorders

The CIDI assessment included six DSM-IV [34] diagnoses analysed in this report: major depressive episode (MDE), manic or hypomania episode (MAN), generalized anxiety disorder (GAD), panic disorder (PD), post-traumatic stress disorder (PTSD), and alcohol use disorder (AUD; including both alcohol abuse and dependence). We selected these six disorders from 12 mental disorders assessed in the WMHJ2 survey [31], while we excluded phobic disorders because these disorders have an onset in an early life [35], dysthymia because the diagnosis requires longer duration criteria (i.e., two or more years) [34] and may not be relevant for this 3-year retrospective recall survey, and drug use disorder because of its very low prevalence in Japan [31]. The hierarchy rule was not applied: for instance, GAD was diagnosed even if MDE was present. For MDE, the exclusion of post-bereavement depression (the criteria E) was not used. This was because of lack of precise information to apply the hierarchy rule, as well as for the sake of simplicity. Prevalence of any of the six mental disorders was labelled as ANY.

All diagnoses were assessed for the respondent’s lifetime, and then, the age of first onset was asked. For mood (MDE and MAN) and anxiety disorders (GAD, PD, and PTSD), respondents from the disaster area were asked additional questions about the cause of the episode and whether the onset was before or after the disaster. Time of onset of each disorder was then classified into before the Great East Japan Earthquake (hereafter the disaster), the same year of the disaster (2011) (1st year), the 2nd year (2012), and the 3rd year (2013) after the disaster.

For disorders that first occurred after the disaster, duration (years) was calculated based on questions on the maximum length of duration for the new onset disorder. If the disorder was active during the 30 days before the survey, the disorder was classified as on-going; otherwise, the respondent was judged as having remitted.

### Disaster experiences

Respondents were asked about three types of disaster experiences: personal injury (any vs. none), bereavement of family/relative(s) or friend(s)/acquaintance(s), and house damage (any vs. none). Most previous studies of post-disaster mental health used one bereavement variable combining bereavement of family and friends [36–39]. In this study, in order to investigate individual and additive effects of bereavement of family and friends, the bereavement was classified into four groups: no bereavement, bereavement of family/relative, bereavement of friends/acquaintances, and both. Perceived radiation risk was determined from responses to a single item: “For several months after the accident of the Fukushima Dai’ichi Nuclear Power Plant, how much were you worried about the possibility that you and your family members were exposed to radioactivity? (Not at all, a little, to some extent, much, or very much)” Those who responded “much” or “very much” were classified as high perceived radiation risk.

### Seeking treatment

The CIDI included a standard set of questions on seeking treatment for problems with mental health and substance [40]. Medical treatment was defined as a visit to psychiatrists or other medical doctors. The proportion of these who sought medical treatment (either psychiatrists or other medical doctors) was calculated among respondents who had a post-disaster mental disorder.

### Demographic variables

Demographic variables assessed during the interview included sex (male vs female), age (20–39, 40–64, and 65+ years old), marital status (married, divorced/separated, widowed, and never married), education (less than high school and high school or higher), and activities of daily living (ADLs) (limited and not limited).

### Statistical analyses

Respondents who had experience of any of the six mental disorders in their lifetime prior to 2011 were excluded from the analyses to investigate the new onset of any of the mental disorders. First, cumulative incidences (i.e., proportions of respondents who had a disorder before a certain point including those who remitted after the onset) of ANY and specific mental disorders among were compared between the shelter sample and the general population sample. The discrete-time proportional hazard model applying the logistic regression [41] was used to estimate odds ratios (ORs) of the incidence in the shelter group compared to the general population, adjusting for sex, age, and education, because the time intervals were discrete. Speed of remission from ANY post-disaster mental disorder was compared between the two samples by using the Kaplan-Meier survival curve (log-rank test for a statistical significance). The median duration (in years) and 95% CI of the individual mental disorders were also calculated.

In the shelter group, sociodemographic and disaster-related factors associated with ANY new onset post-disaster mental disorder were investigated with using the discrete-time proportional hazard model. Disorder-specific analyses were conducted for individual mental disorders with 10 or more cases. Among respondents who developed post-disaster mood or anxiety disorders in the shelter group, the association between each of socio-demographic and disaster-related factors and remission from the mental disorder was investigated by using the Kaplan-Meier method with log-rank test.

All variables used in these analyses had no missing response. All statistical analyses were conducted with IBM SPSS Statistics version 22.

## Results

### Descriptive characteristics

A total of 1089 long-term residents of the 13 shelters in the disaster area (the shelter group) were interviewed, including 242 (55.4%) out of 437 in Iwate, 329 (55.6%) out of 592 in Miyagi, and 518 (44.0%) out of 1178 in Fukushima. The overall response rate for shelter respondents was 49.4%. The response rate for the general population was 46.1% (852 out of 1850). Respondents from the shelters were more likely to be female, older, widowed, have low levels of education, and report disaster-related experiences compared to the general population (Table 1). Shelter respondents had significantly lower rates of most pre-disaster disorders compared to the general population.

**Table 1:** Characteristics of the shelter group and the general population sample 3 year after the Great East Japan Earthquake in 2011

	Shelter group (*n* = 1089)		General population in east Japan (*n* = 852)		*p* for difference^a^
	*n*	%	*n*	%	
Sex (females)	667	61.2	438	51.4	< 0.010
Age, years					
20–39	100	9.2	233	27.3	< 0.001
40–64	357	32.8	427	50.1	
65+	632	58.0	192	22.5	
Marital status					
Married	628	57.7	615	72.2	< 0.001
Divorced/separated	75	6.9	46	5.4	
Widowed	266	24.4	39	4.6	
Never married	120	11.0	152	17.8	
Education (high school or higher)	653	60.0	788	92.5	< 0.001
ADL (limited)	57	5.2	1	0.1	< 0.001
Disaster experiences					
Own injury (any)	53	4.9	1	0.1	< 0.001
Bereavement during the disaster					
None	450	41.3	835	98.0	< 0.001
Family/relative	313	28.7	5	0.6	< 0.001
Friend/acquaintance	128	11.8	12	1.4	< 0.001
Both	198	18.2	–	–	< 0.001
House damage (any)	877	80.5	23	2.7	< 0.001
Radiation anxiety (high)	326	29.9	118	13.8	< 0.001
Lifetime prevalence of mental disorders before the disaster					
Any^b^	79	7.3	157	18.4	< 0.001
Major depressive episode	13	1.2	32	3.8	< 0.001
Manic episode	1	0.1	2	0.2	0.587
GAD	6	0.6	16	1.9	0.008
Panic disorder	4	0.4	4	0.5	0.736
PTSD	4	0.4	3	0.4	1.000
Alcohol use disorder	64	5.9	123	14.4	< 0.001

^a^Chi-square test for age and marital status; otherwise, Fisher exact test

^b^Any of six mental disorders including major depressive episode, manic episode, generalized anxiety disorder (GAD), panic disorder, post-traumatic stress disorder (PTSD), and alcohol use disorder

### Incidence and remission of post-disaster mental disorders in the shelter group and the general population

In the shelter group and the general population, 3.3 and 0.7% of respondents, respectively, met criteria for ANY mental disorder in 2011, the year the disaster happened (Table 2). The three-year cumulative incidence until 2013 was 5.6 and 2.7%, respectively. MDE was the most common disorder in the both samples. The three-year cumulative incidence of ANY mental disorder was significantly higher in the shelter group than in the general population (*p* = 0.010 for crude analysis and *p* = 0.002 after adjusting for sex, age, and education). In terms of specific disorders, compared to the general population, the shelter group had significantly higher cumulative incidences of MDE, GAD, and PTSD (*p* < 0.01), but not MAN, PD, or AUD (*p* > 0.05).

**Table 2:** Cumulative incidence of mental disorders among respondents without pre-disaster mental disorders following the Great East Japan Earthquake 2011^a^

	ANY % (SE)	MDE % (SE)	MAN % (SE)	GAD % (SE)	PD % (SE)	PTSD % (SE)	AUD % (SE)
Shelter group (*n* = 1010)^b^							
Post disaster year 1	3.3(0.6)	2.9(0.5)	0.2(0.1)	1.4(0.4)	0.1(0.1)	0.6(0.2)	0.1(0.1)
Post disaster year 2	4.8(0.7)	4.1(0.6)	0.2(0.1)	1.9(0.4)	0.1(0.1)	1.0(0.3)	0.2(0.1)
Post disaster year 3	5.6(0.7)	4.3(0.6)	0.2(0.1)	2.3(0.5)	0.2(0.1)	1.0(0.3)	0.4(0.2)
General population (*n* = 695)^b^							
Post disaster year 1	0.7(0.3)	0.3(0.2)	0(−)	0.1(0.1)	0(−)	0(−)	0.4(0.2)
Post disaster year 2	1.3(0.4)	0.6(0.3)	0(−)	0.1(0.1)	0(−)	0(−)	0.7(0.3)
Post disaster year 3	2.7(0.6)	1.4(0.5)	0.1(0.1)	0.3(0.2)	0(−)	0(−)	1.2(0.4)
Log-rank p for group difference	0.010	0.006	0.521	0.001	0.240	0.006	0.072
Cox hazard model adjusted for sex, age, and educational attainment:							
Adjusted hazard ratio	2.23	2.46	4.65	11.13	NC	NC	0.60
95%CI	(1.33–3.75)	(1.27–4.76)	(0.45–48.09)	(2.55–48.65)			(0.17–2.08)
*p*	0.002	0.007	0.299	0.001			0.421

^a^Percentage (%) and the standard error (SE) were presented

^b^Respondents with any disorder before the disaster were excluded. Any disorder includes any of six mental disorders including major depressive episode (MDE), manic episode (MAN), generalized anxiety disorder (GAD), panic disorder (PD), post-traumatic stress disorder (PTSD), and alcohol use disorder (AUD)

*NC* not calculated

**Fig. 1:**
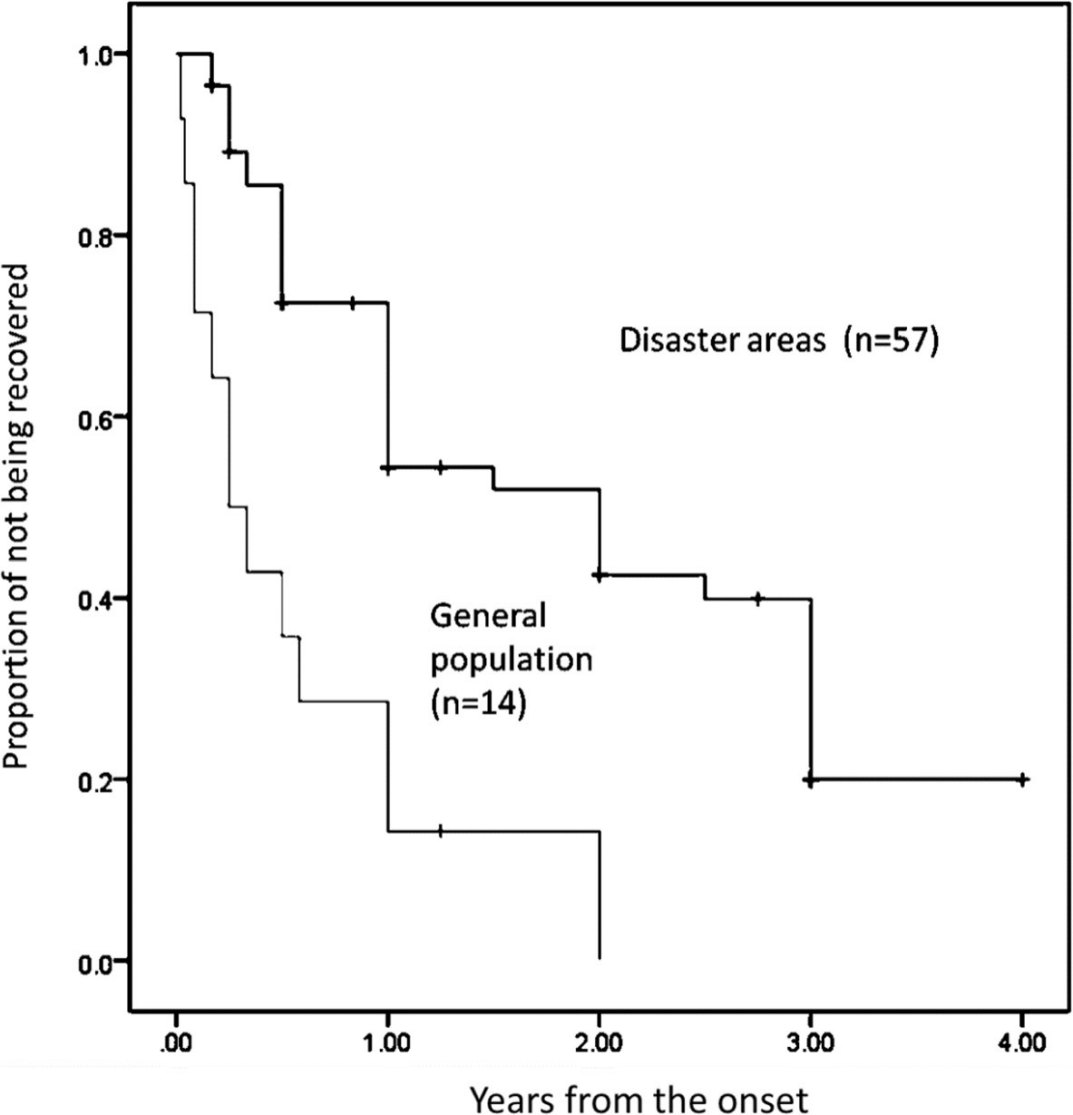
Remission from post-disaster DSM-IV mood and anxiety disorders in the shelter group and in the general population. A significant difference in the remission was observed between respondents in the shelter group and the general population (Logrank test, *p* < 0.001)

The proportion of individuals who had not remitted from ANY post-disaster mood and anxiety disorders (excluding alcohol use disorder) was higher in the shelter group (*n* = 57) than in the general population (*n* = 14) (log-rank test, chi-square = 17.9, df = 1, *p* < 0.001) (Fig. 1). The median durations of post-disaster ANY were 2.00 years (standard error [SE], 0.36) and 0.25 years (SE, 0.10) in the shelter group and the general population, respectively.

### Seeking treatment for post-disaster mental disorders

The proportions of individuals who visited psychiatrists or other physicians among people with post-disaster ANY were greater in the shelter group (42.6%, 26 of 61 cases) than in the general population (17.4%, 4 of 23 cases) (Fisher exact test, *p* = 0.011). The proportions of those seeking treatment for MDE were 39.5% (17 of 43 cases) for the shelter group and 30.8% (4 of 13 cases) for the general population (Fisher exact test, *p* = 0.339). The proportion of treatment for PTSD (9.1%) was lower than for the other disorders in the shelter group, while no comparison was possible between the two groups because of no post-disaster cases with PTSD in the general population. None of respondent with post-disaster AUD (*n* = 5 for the disaster areas; *n* = 9 for the general population) had visited psychiatrists or other physicians.

### Disaster risk factors associated with onset and remission of post-disaster mental disorders in the shelter group

In the shelter group, being divorced/separated (*OR* = 3.00, 95%*CI* = 1.35–6.68, *p* = 0.007), personal injury (*OR* = 6.10, 95% *CI* = 2.97–12.55, *p* < 0.001), and bereavement of both family/relative and friend (*OR* = 2.73, 95% *CI* = 1.13, 6.60, *p* = 0.026) were significantly associated with post-disaster onset of ANY disorder (Table 3). In the disorder-specific analyses (data available upon request), it was found that female sex (*OR* = 2.41, 95%*CI* = 1.12–5.17, *p* = 0.024), being never married (*OR* = 4.05, 95%*CI* = 1.42–11.53, *p* = 0.009), personal injury (*OR* = 8.84, 95%*CI* = 3.91–20.00, *p* < 0.001), and bereavement of both family/relative and friends (*OR* = 3.41, 95% *CI* = 1.23–9.42, *p* = 0.018) were significantly associated with the onset of MDE. Personal injury (*OR* = 8.65, 95% *CI* = 1.95–38.42, *p* = 0.005) and bereavement of both family/relative and friend (*OR* = 22.53, 95% *CI* = 1.67–303.83, *p* = 0.019) were significantly associated with PTSD. Personal injury was the only disaster risk factor significantly associated with the onset of GAD (*OR* = 5.98, 95% *CI* = 2.08–17.21, *p* = 0.001).

**Table 3:** Factors associated with post-disaster mental disorders in the shelter group during 3 years after the Great East Japan Earthquake 2011^a^

Variables	OR^b^	95%CI (low to high)		p
Sex (female)	0.69	0.38	1.27	0.235
Age, years				
20–39	1.00			
40–64	1.56	0.57	4.31	0.388
65+	1.38	0.46	4.17	0.570
Education (high school or higher)	1.12	0.61	2.04	0.721
Marital status				
Married	1.00			
Divorced/separated	3.00	1.35	6.68	0.007
Widowed	0.90	0.45	1.82	0.778
Never married	1.90	0.75	4.82	0.174
Current ADL (limited)	0.55	0.12	2.39	0.423
Personal injury (yes)	6.10	2.97	12.55	< 0.001
Bereavement during the disaster				
None	1.00			
Family/relative	0.94	0.45	1.95	0.867
Friend/acquaintance	1.87	0.80	4.37	0.150
Both	2.73	1.13	6.60	0.026
Home damage (yes)	2.12	0.90	5.00	0.085
Radiation risk perception (high)	1.46	0.80	2.68	0.217
Study area (prefecture)				
Iwate	1.00			
Miyagi	0.80	0.31	2.04	0.638
Fukushima	1.75	0.72	4.26	0.216

^a^ Discrete proportional hazard model was used. Among 1010 respondents, 61 developed any of six post disaster mental disorders (major depressive episode, manic episode, generalized anxiety disorder, panic disorder, PTSD, and alcohol use disorder)

^b^*OR* odds ratio

None of the demographic and disaster-related factors were significantly associated with the remission from post-disaster mood and anxiety disorders among 57 respondents who had any post-disaster mood and anxiety disorder (Kaplan-Meier method with the log-rank test, *p* > 0.05). (data available upon request).

### Post-hoc power analysis

For the comparison of the three-year cumulative incidences of post-disaster mental disorders (z-test), we observed an about two-fold difference between the shelter sample (5.6% among 1010 respondents) and the general population (2.7% among 695 respondents). The statistical power to detect the difference with two-tailed *p* = 0.05 was estimated as 0.85.For the comparison of the remission of post-disaster mental disorders (log-rank test), for instance, at one-year, we observed median survival durations in the shelter sample (2.00 year among 57 respondents) and the general population (0.25 years among 14 respondents). An estimation of the statistical power to detect the difference with two-tailed *p* = 0.05 was 0.91.

## Discussion

Among the survivors living in temporary housing for 3 years or longer after the 2011 Great East Japan Earthquake, 3.3% experienced a new onset of a common mental disorder in the first year (in 2011), five times higher than the general population. However, the incidence levelled off in the second and third years and became similar to that of the general population. On the other hand, remission from post-disaster mood and anxiety disorders was prolonged in the shelter group compared to the general population, even though the proportion seeking medical treatment was greater in the shelter group. These findings suggest that poor mental health among disaster survivors who stayed in temporary housing for several years is in part attributable to slow remission from common mental disorders despite greater use of medical services.

About 1 in 18 respondents living in the shelter group developed a new mental disorder during the three-year period after the disaster. This three-year incidence was about two times higher than in the general population. For individual disorders, the incidences were significantly higher for MDE, GAD and PTSD, but not for AUD, as has been reported in some previous post-disaster studies [4, 6–11] . The first-year incidence in the shelter group was particularly higher compared to that in the general population. However, there were few delayed onset cases in the second and third years. This is consistent with previous post-disaster studies of mixed samples of displaced and non-displaced survivors [6, 8, 10, 11] . The present finding replicates our previous finding on incident suicide ideation from the same sample [28], and extends this pattern into common mental disorders.

In the shelter group, those who had been injured were more likely to develop ANY and specific mental disorders (MDE, GAD, and PTSD). Bereavement of both family and friends was significantly associated with ANY as well as MDE and PTSD. These findings are consistent with previous symptom-based studies [1, 4, 38, 42]. In the shelter group, the association of bereavement of friends with mental disorders was slightly larger than that of family. This may be partly because most bereavement cases lost older family members [43] and thus might have accepted their deaths more readily. In addition, respondents who lost their family may receive more sympathy and support from the community.

The present study found that remission from post-disaster mood and anxiety disorders was delayed in the shelter group compared to the general population. The median duration of morbidity was 2.0 years in the shelter group. Previous studies of survivors who were relocated to temporary housing for two or 3 years consistently reported elevated levels of psychological distress after the Great East Japan Earthquake [24–27] . Poor mental health in this group may partly be attributable to the slow time to remission from post-disaster common mental disorders. Possible factors underlying the prolonged remission may include reduced physical activity and impaired physical function in this group [44, 45] that may prevent a faster remission from depression and anxiety disorders [46]. The other possibility is that remission was delayed because of deteriorating physical health due to conditions of the temporary housing [24, 47]. Lower job opportunities among people living in the temporary housing may also delay the remission from mental disorders [48]. Other disadvantages in social, economic, and working life may also affect remission among people who still lived in the temporary housing 3 years after the disaster compared to those who left earlier. Future studies need to disentangle the various stressors inherent in temporary housing to identify adversities that are modifiable.

The proportion of respondents seeking medical treatment among those who suffered a post-disaster mental disorder was higher in the shelter group than in the general population. In a previous report, survivors of disasters were reluctant to use mental health services [30]. The present finding is attributable to post-disaster mental health measures taken by the government, local communities, and non-profit organizations [43, 49, 50]. For instance, in most disaster areas, community health nurses and other field workers/volunteers closely monitored the mental and physical health of people living in temporary housing after the disaster, and referred them to medical treatment if they observed a problem. It is puzzling that the shelter group experienced a prolonged remission from mental disorders, despite a higher treatment rate. However, it is difficult to draw conclusions on the effectiveness of medical treatment from this observational study. Further well-designed research is needed to explore its effectiveness. Also, the treatment rate was lower for PTSD and AUD. Associated behavioural characteristics, such as social withdrawal, may prevent people with these disorders from accessing medical treatment. In addition to providing medical treatment, psychological treatment and psychosocial support including and group peer support may be beneficial.

The present study did not find disaster-related factors that were significantly associated with the delay in remission of post-disaster mood and anxiety disorders in the shelter group. However, this may be due to the small number of respondents who developed these disorders in the shelter group. It may have a merit to investigate factors associated with the remission separately from factors associated with the incidence in disaster survivors under different social and living situations. Factors associated with the remission should be investigated further in a large scale prospective study of disaster survivors.

### Limitations

Several limitations should be noted in the context of the present study. First, the findings should be generalized with caution since study sites of the shelter group were based on the availability of local investigators and the willingness of the director to participate in the study. Thus, the shelters could not be selected randomly. However, the surveys covered all three prefectures affected by the disaster. Second, the selection biases should be considered. The cumulative incidence in the present study may be underestimated because some residents who developed severe mental disorders may have been hospitalized or institutionalized. The cumulative incidence may also have been overestimated because disaster survivors with socioeconomic disadvantages may stay in temporary housing longer, which could result in a higher incidence. In addition, the response rate of the study was not high. A previous study reported that the response rate may not be strongly associated with the prevalence estimates [51]. However, it was possible that residents in temporary housing who had mental disorders were reluctant to participate in the study. The cumulative incidences could be lower than the whole shelter samples and community residents because we selected respondents who had no mental disorder before the disaster. Third, the findings are subject to recall bias. Pre-disaster prevalence of mental disorders was much lower for the shelter group than the general population, possibly because the affected areas were located in rural coastal areas where the prevalence of mental disorders is lower in general [52]. However, a reporting bias due to social desirability may affect the responses to CIDI questions among the shelter sample. The differential reporting of the pre-disaster prevalence and post-disaster incidence of mental disorders in the two samples may affect the findings. Fourth, respondents in the shelter group may report post-disaster mental disorders more frequently because the disorder onset was closely related to a memory anchor, that is, the disaster. This may have been reflected in the low prevalence of pre-disaster mental disorders in the disaster area sample. Furthermore, in analyses of factors associated with mental disorders, respondents with mental disorders may have reported more disaster-related events, owing in part to cognitive issues. Fifth, the determination of the year of onset of mental disorders depended on the information on current age and age of onset. The accuracy could thus be off by 1 year in some cases. However, the interview contained specific probes to maximize accuracy of dating onset of each mental disorder. Sixth, possible confounding factors should be considered. Sex, age, and educational attainment were adjusted in the analyses, but it may not be enough to make the samples from the disaster areas and the general population comparable. Indicators of current socioeconomic status, such as household income and employment, were not adjusted. Pre-disaster socioeconomic indicators other than education were not measured in this study. Future studies should include this information. Finally, the sample size and the number of cases of individuals who developed mental disorders were small. Thus, some of the observed differences should be interpreted carefully, and the study may have been underpowered to detect significant differences especially for the individual disorders. Likewise, we could not compare the remission between the two groups for each disorder, which made the interpretation of the findings difficult. A large scale prospective study of disaster survivors is needed to overcome these weaknesses of the present study.

## Conclusions

The onset of common mental disorders increased in the first year, but then levelled off in the following years among residents in temporary housing after the triple disaster in the Tohoku area of Japan in 2011. The overall remission from post-disaster mood and anxiety disorders was slower in the shelter residents than in the general population. Post-disaster mental health service should consider a greater incidence in the first year and prolonged remission of mental disorders among survivors with a long-term stay in temporary housing after a disaster.

## Supplementary information

**Additional file 1:.**

## Data Availability

The datasets used and analysed during the current study are available from the corresponding author on reasonable request.

## Abbreviations

PTSD: Post-traumatic stress disorder
DSM-IV: Diagnostic and Statistical Manual of Mental Disorders, fourth edition
WMHJ2: The World Mental Health Japan Second Survey
CIDI: Composite International Diagnostic Interview
MED: Major depressive episode
MAN: Manic or hypomania episode
GAD: Generalized anxiety disorder
PD: Panic disorder
AUD: Alcohol use disorder
ANY: Any of the six mental disorders
ADLs: Activities of daily living
OR: Odds ratio
SE: Standard error
