# Supplementary material for: Onset and remission of common mental disorders among adults living in temporary housing for three years after the triple disaster in Northeast Japan: comparisons with the general population
Source: BMC Public Health. 2020 Aug 20;20:1271. doi: 10.1186/s12889-020-09378-x (PMC7441677; doi:10.1186/s12889-020-09378-x)
Supplement: Supplementary file 1 — Additional file 1:. [file 12889_2020_9378_MOESM1_ESM.pdf]

HARVARD MEDICAL SCHOOL

DEPARTMENT OF HEALTH CARE POLICY

180A Longwood Avenue
Boston, MA 02115-5899

TEL: (617) 432-2594
FAX: (617) 432-3588

August 10, 2020

Professor Norito Kawakami is a member of the WHO World Mental Health Survey (WMH) consortium and Principal Investigator of the WMH Japan surveys.

He has completed the required training to use the CIDI 3.0 and had permission from me and the WMH Executive committee to use the CIDI 3.0.

Yours sincerely,

Ronald C. Kessler, McNeil Family Professor of Healthcare Policy
